# Herbal Supplement Extends Life Span Under Some Environmental Conditions and Boosts Stress Resistance

**DOI:** 10.1371/journal.pone.0119068

**Published:** 2015-04-16

**Authors:** Bryant Villeponteau, Kennedy Matsagas, Amber C. Nobles, Cristina Rizza, Marc Horwitz, Gregory Benford, Robin J. Mockett

**Affiliations:** 1 Genescient Inc., Fountain Valley, California, United States of America; 2 University of California, Irvine, California, United States of America; 3 University of South Alabama, Mobile, Alabama, United States of America; CSIR-Central Drug Research Institute, INDIA

## Abstract

Genetic studies indicate that aging is modulated by a great number of genetic pathways. We have used *Drosophila* longevity and stress assays to test a multipath intervention strategy. To carry out this strategy, we supplemented the flies with herbal extracts (SC100) that are predicted to modulate the expression of many genes involved in aging and stress resistance, such as mTOR, NOS, NF-KappaB, and VEGF. When flies were housed in large cages with SC100 added, daily mortality rates of both male and female flies were greatly diminished in mid to late life. Surprisingly, SC100 also stabilized midlife mortality rate increases so as to extend the maximum life span substantially beyond the limits previously reported for D. melanogaster. Under these conditions, SC100 also promoted robust resistance to partial starvation stress and to heat stress. Fertility was the same initially in both treated and control flies, but it became significantly higher in treated flies at older ages as the fertility of control flies declined. Mean and maximum life spans of flies in vials at the same test site were also extended by SC100, but the life spans were short in absolute terms. In contrast, at an independent test site where stress was minimized, the flies exhibited much longer mean life spans, but the survival curves became highly rectangular and the effects of SC100 on both mean and maximum life spans declined greatly or were abolished. The data indicate that SC100 is a novel herbal mix with striking effects on enhancing *Drosophila* stress resistance and life span in some environments, while minimizing mid to late life mortality rates. They also show that the environment and other factors can have transformative effects on both the length and distribution of survivorship, and on the ability of SC100 to extend the life span.

## Introduction

Many studies indicate that aging is modulated by a large number of aging-related genes that often boost stress resistance [1–7]. This multipath nature of aging suggests that many genetic patterns must be altered simultaneously for a successful intervention into the aging process. In devising our longevity experiments, our overall aim was to test the hypothesis that aging in *Drosophila* can be significantly slowed using an environmental intervention that acts on multiple longevity genes and boosts stress resistance. We have tried to target several of the known longevity genes, as well as stress resistance pathways such as oxidative stress, heat stress, starvation stress, environmental stress, and excessive neuronal activation. As we detail below, the four herbal extracts in SC100 provide a diverse set of bioactive compounds, which appear to act on various pathways such as activators of telomerase, inhibitors of mTOR, polyphenolic antioxidants, insulin sensitizers, stem cell activators, parasympathetic agonists, and stress response genes.

Our initial stress and longevity experiments were done in large cages with one strain of flies, as this allowed large numbers of flies (500 per cage) to be followed with the minimum of handling. Nevertheless, we repeated the longevity experiments in vials using different fly strains, laboratories, and other conditions. The wide range of conditions used permitted us to explore the considerable role of environment in affecting life span.

To develop potential oral antiaging supplements, we initially set out to identify nutraceutical or drug compounds that would target as many of the complementary longevity and stress pathways as possible and thereby extend *Drosophila* life span. Unfortunately, with the exception of the drug lithium, none of the single compounds that we initially tested appeared to significantly extend fly life span in our longevity or stress resistance screens. The lithium results and some of our negative single substance data were published [8]. The typically poor longevity effects of single oral compounds suggested that many single substance therapeutics directed to a single target may not significantly extend life span.

We began testing mixtures of medicinal herbal extracts, as these have had a long history of clinical success in Chinese and Indian traditional medicine and are known to have a wide spectrum of positive effects in humans [9–13]. To affect as many longevity genes as possible, we focused on complementary herbal extracts that have antioxidant, anti-inflammatory, and metabolic properties (known factors in driving aging and stress) along with a positive effect on longevity genes and a history of use in traditional herbal medicine to treat a wide spectrum of diseases. A combination of four herbal extracts (SC100) containing *Astragalus membranaceus* root, *Pterocarpus marsupium* bark, pine bark oligo-proanthocyanidins, and L-theanine was more effective than any single substance or other combination of substances that we tested in our preliminary stress resistance screens in *Drosophila*.

Aside from our longevity screening results, some of the published data on longevity genes, as detailed below, also suggest that the 4-herb mix of SC100 might work synergistically to extend fly longevity. For example, stem cells are important for maintaining *Drosophila* intestines and for high fertility rates. The major herbal extract in SC100 comes from the Chinese medicinal herb *Astragalus membranaceus* and *Astragalus* extracts are well known to promote stem cells in several model systems [14–18]. Moreover *Astragalus* extracts are known to have favorable cardioprotective and angiogenic effects in rats, activate the expression of VEGF (Vascular Epithelial Growth Factor), and inhibit mTOR (mammalian Target Of Rapamycin) expression [19,20]. Low VEGF levels are linked to dementia [21–23] and aging [24–26], while the mTOR gene is a well-known modulator of mammalian life span [27]. The stem cell, VEGF, and TOR altering components of *Astragalus* may explain some of the SC100-induced effects on life span.

The second major herbal extract in SC100 is *Pterocarpus marsupium* bark (also called Indian Kino Tree), which has been standardized for a high content of the resveratrol analog pterostilbene. Pterostilbene is thought to be the key active ingredient in *Pterocarpus marsupium* and is much more efficacious than resveratrol in reversing cognitive deficits in aged rats [28]. Pterostilbene has also been shown to reduce colon tumors, pro-inflammation cytokines, and Alzheimer pathology in rodents [29,30]. *Pterocarpus marsupium* bark also contains the resveratrol analog marsupsin, which significantly lowered blood glucose in hyperglycemic rats at a level comparable to the antidiabetic drug metformin [31]. Another resveratrol analog in *Pterocarpus* bark is pterosupin, which lowers serum triglyceride and LDL cholesterol in hyperlipidemic rats [32].

The third herbal extract in SC100 is Pine Bark Extract (PBE), which was purified to 85% proanthocyanidins. PBE improves endothelial function via activation of endothelial nitric oxide synthesis [33] and is reported to help chronic venous insufficiency [34,35]. PBE also has some anti-inflammatory potential, as it inhibits matrix metalloproteinase 9 (MMP-9) and NF-Kappa B activation [36].

The fourth herbal extract in SC100 is L-Theanine, which is a unique neuroprotective amino acid found in green tea that crosses the blood-brain barrier. L-Theanine has a structure similar to glutamate, which is a neurotransmitter related to memory, and binds to the GABA receptors in neurons. Even doses as low as 1 mg of L-Theanine per kg of body weight reduce the sizes of cerebral infarcts following middle cerebral artery occlusion in mice [37]. L-Theanine also attenuated neurotoxicity of rotenone and dieldrin-induced DNA fragmentation and apoptotic death in cultured neural cells [38], as well as L-glutamate-induced amyloid beta neurotoxicity [39]. Moreover, L-Theanine is reported to facilitate neurogenesis in the hippocampus of rats following treatment, leading to enhanced memory [40]. As to its antiaging effects, L-Theanine can suppress the shortened life span and learning impairment of senescence accelerated mice under stress [41].

## Results

### SC100 Extended *Drosophila* Mean and Maximum Life Span in Cages: CA Site

**Fig 1:**
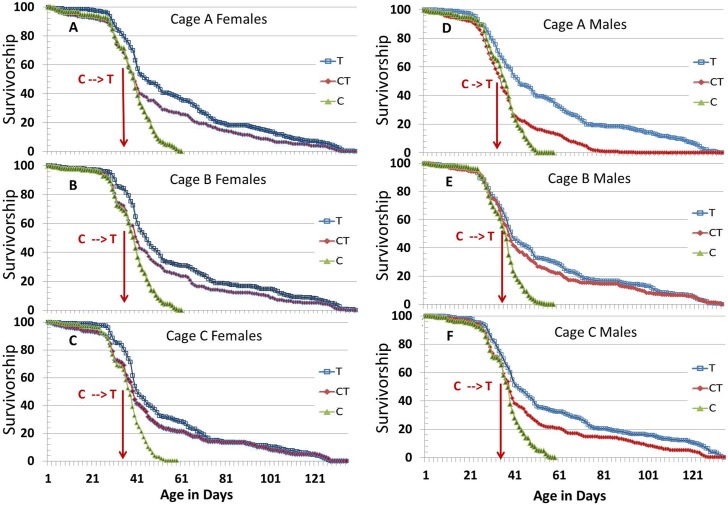
Life span of *Drosophila* in cages with or without SC100 treatment. Nine independent cages were used in 9 simultaneous longevity assays, with each cage having 250 male and 250 female B4 flies at 25°C. Female or male sets of 3 cages each (Cages A, B, or C) are shown with the Survivorship (percent survival) on the Y axis and the Adult Age in days on the X axis. These data show Survivorship for: **C**) Control Untreated flies (green triangles), **T**) SC100 Treated flies (blue open squares), and **CT**) Control/Treated flies that were switched to SC100 treatment on day 36 of the assay (red diamonds). For each treatment, females in Fig 1A, 1B, and 1C were housed together with males in Fig 1D, 1E, and 1F, respectively.

In our large cage experiments, we used three cages with 250 males and 250 females per cage for each longevity screen, so that the longevity experiments were all done in triplicate cages. The three treatment conditions were: C) Control untreated flies; T) SC100 treated flies; and CT) Control/Treated flies that were switched from control conditions to SC100 treatment on day 36 of the longevity assay. Fig 1 shows female (Fig 1A–1C) and male (Fig 1D–1F) fly survivorship for each of the three replicate treatment conditions (Cages A, B, and C).

As can be observed in Fig 1, SC100 extended *Drosophila* life span and generated significantly lower mortality at later population ages. Similar life span extension occurred in both females and males. Fig 1 shows that untreated Control flies (green solid triangles) were all dead before 60 d, while the SC100 treated samples (blue open squares) did not die off completely until around 134 d. Surprisingly, SC100 worked nearly as well when the flies were on the control regimen for 36 d and then shifted to SC100 at 36 d (red solid diamonds).

**Fig 2:**
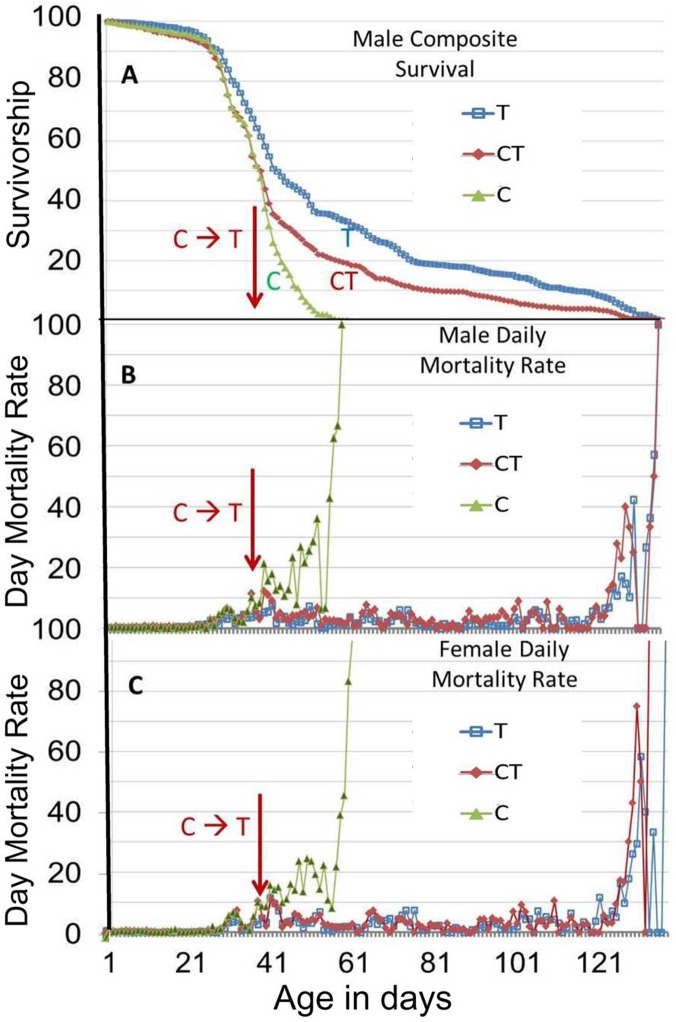
Male and female mortality rate changes in cages with or without SC100 treatment. (**A**) These data are from the same longevity assays shown in Fig 1, wherein nine independent cages were used, with each cage having 250 males and 250 female B4 flies. To correlate mortality rates with the longevity assays, the male longevity data in the 3 sets of cages of Fig 1D, 1E, and 1F were pooled and composite average values were taken on each day for: *C*) Control untreated male flies (green triangles), **T**) SC100 treated male flies (blue squares), and **CT**) Control/Treated flies that were switched to SC100 treatment on day 36 of the assay (red diamonds). (**B**) and (**C**) show composite daily mortality rates for male flies (B) and female flies (C), which were determined by averaging the daily mortality rates from the three sets of cages for each treatment or control.

Another way to look at these longevity data for flies in cages is to look at the daily mortality rate (Fig 2). The control flies (green triangles) had an increase in their daily mortality rate at around 28 d and their mortality rate gradually increased until 54–58 d, when the daily mortality rate spiked as the last flies died. In both the lifetime SC100 treated flies (T, open blue squares) and the mid-life SC100 treated flies (CT, red diamonds), the mortality rate did not increase substantially until around 120 d, ending in a spike at 120–134 d. The midlife SC100 treated flies actually decreased their mortality rate several days after SC100 treatment commenced at 36 d and continued with a low stable mortality rate until the mortality spike at 120–134 d (Fig 2B and 2C). Thus, *SC100 stabilized the midlife mortality rate in the flies and that may play a role in how SC100 extended maximum life span of a major fraction of the fly population*.

SC100 had a highly significant effect on mean longevity. Table 1 gives a summary of mean longevities for B4 flies in cages in days for the Control (C), SC100-treated (T), and Control shifted to SC100-treated (CT) flies, along with their 95% confidence intervals. From Table 1 we can conclude that lifelong SC100-treated and midlife SC100-treated flies both yielded significantly higher mean longevities than the Control flies. In comparison to the untreated Control, the lifelong SC100-treated flies had a 49% increase in mean life span, while the midlife SC100-treated flies had a 27% increase in mean life span. Males and females did not have a significant difference in longevity.

**Table 1:** Mean *Drosophila* life spans in cages and contrasts with control or differing SC100 treatments.

**Population/Contrast**	**Longevity (days)**	**±95% CI**
Control (C)	37.6	±2.5
Control SC100 (CT)	47.6	±3.1
SC100 (T)	56.1	±3.1
CT-C Increase	10.0	±3.1
T-C Increase	18.6	±3.1
T-CT Increase	8.6	±3.1

Mean life spans for Control, Control switched to SC100 treated at 36 days, and SC100 treatments throughout the life span are given in days. The ±95% CI is the Confidence Interval (CI) in days.

SC100 had particularly strong effects on maximum longevity, which is measured as the number of days lived by the top 10% or 5% of survivors. Table 2 gives the data for the C, T, and CT flies. Based on the confidence intervals, *there was a highly significant doubling of maximum life span on SC100 treatment* and this was true even in the case where the treatment started at midlife (36 days).

**Table 2:** Maximum *Drosophila* life spans in cages for the top 10% and 5% of surviving flies for control and differing SC100 treatments.

**Population**	**Mean Top 10% Survival (days)**	**±95% Cl**	**Mean Top 5% Survival (days)**	**±95% Cl**
Control (C)—Male	46.9	±2.0	50.1	±3.7
Control (C)—Female	47.5	±2.2	50.5	±4.2
Control SC100 (CT)—Male	97.2	±2.0	121	±5.0
Control SC100 (CT)—Female	94.6	±6.4	118	±9.2
SC100 (T)—Male	114	±17.8	125	±6.3
SC100 (T)—Female	106	±2.1	122	±5.9

The ±95% CI is the Confidence Interval (CI) in days.

### Gompertz Parameters *A* and α

The effect of these treatments on the age-specific mortality (or survival) patterns can be examined in more detail by looking at the effect of each treatment and sex on the two parameters of the Gompertz equation, which is determined by the *rate of aging parameter* **α** and the *age-independent parameter* ***A***. Table 3 gives the estimated ***A*** and **α** parameter values. From this table it is clear that the Control flies had one order of magnitude lower age-independent mortality parameter ***A*** than either the lifelong SC100-treated flies or the midlife-SC100-treated flies. Conversely, the rate of aging **α** parameter was much higher in the Control than it was for the two SC100 treatments.

**Table 3:** Estimated Gompertz parameter values in cages from a non-linear mixed effects model.

**Parameter**	**Sex**	**Treatment**	**Estimate**
***A***	Male	Control	0.000484
		Control-> SC100	0.004720
		SC100	0.008280
	Female	Control	0.000481
		Control-> SC100	0.006340
		SC100	0.005920
**α**	Male	Control	0.1430
		Control-> SC100	0.0529
		SC100	0.0195
	Female	Control	0.1400
		Control-> SC100	0.0369
		SC100	0.0294

Table 4 compares the C, T and CT treatments to see whether any of the differences in Table 3 were significant. In every case, the C group had a significantly smaller ***A*** parameter than either the T or TC flies. Conversely, the **α** parameter was significantly greater in the C than in either T or CT flies for both male and female flies (*P*<10^–16^). These are unique observations, as most environmental treatments that increase longevity do so by decreasing the age-independent parameter ***A*** and have little effect on **α**. No treatment has been published previously that has such a pronounced effect on **α** [42–44].

**Table 4:** Treatment contrasts in cages for the two Gompertz parameters are highly significant.

**Parameter**	**Sex**	**Contrast**	**Estimate**	**±95% CI**
***A***	Male	CT-C	0.00423	±0.00069
		T-C	0.00780	±0.00069
	Female	CT-C	0.00586	±0.00073
		T-C	0.00540	±0.00061
**α**	Male	CT-C	-0.0902	±0.013
		T-C	-0.1240	±0.013
	Female	CT-C	-0.1030	±0.014
		T-C	-0.1100	±0.012

The ±95% CI is the Confidence Interval (CI). All results are highly significant with *P*<10^–16^ for all contrasts in cages.

### SC100 Extended the Short Life Span of *Drosophila* Housed in Vials: CA Site

**Fig 3:**
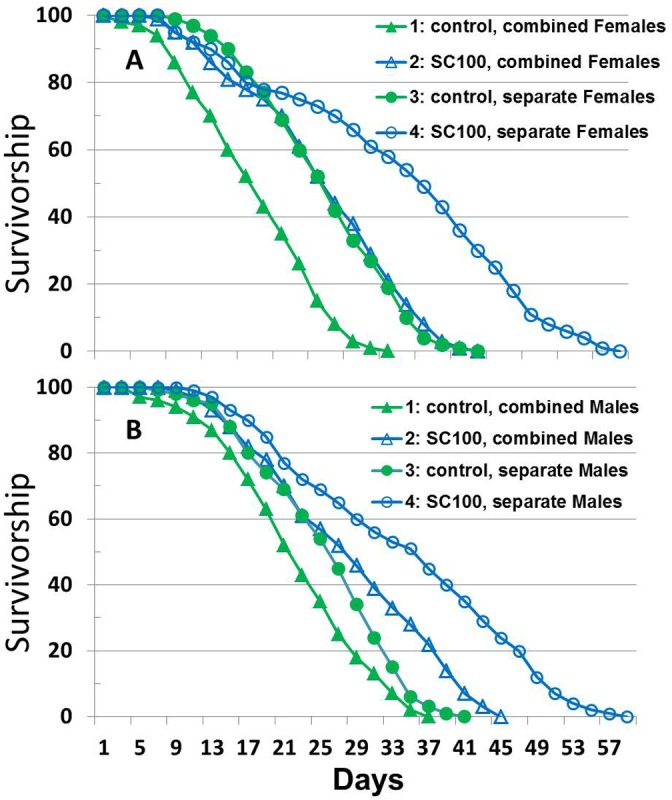
Life span of male and female *Drosophila* in single sex or combined sex vials with or without SC100 treatment: CA site. 3A (Females) and 3B (Males) show the survivorship curves for female and male B4 flies, respectively, where 4 flies of the same sex or 2 flies of each sex were housed at 25°C in each test vial, with 100 flies for each sex in the control or SC100-treated samples. In (**A**), the data for females are shown: Control females were housed in separate vials from males (solid green circles) versus housing of females with males in combined vials (solid green triangles); SC100 treated female flies are housed separate from males (open blue circles) or combined with males (open blue triangles). In (**B**), the data for males are shown: Control males were housed in separate vials from females (solid green circles) versus housing of males with females in combined vials (solid green triangles); SC100 treated male flies were housed separate from females (open blue circles) or combined with females (open blue triangles).

The 500 fly cage longevity screening assay described above is not used as commonly as small test vial experiments where the *Drosophila* are tightly confined as to space and unable to fly. In most test vial fly assays, male and female flies are separated into different vials, as it is well known that sexual activity shortens fly life span. Since the test tube-like vial environment is very different from the cage environment, we tested SC100 on flies in vials combined with or separated from the opposite sex. Fig 3A and 3B show the survivorship curves for females and males respectively, where 4 flies of the same sex or combined sexes were housed in each test vial with 100 flies for each sex in each control and 100 flies for each sex in each of the SC100-treated samples.

As expected, Fig 3A shows that female control flies had longer mean or maximum life spans if housed in separate vials from males (solid green circles) versus maintaining females with males (solid green triangles). SC100 treatment extended mean and maximum life span of the females, whether they were housed separate from males (open blue circles) or combined with males (open blue triangles). Fig 3B shows nearly the same results for males housed with only other males or in combination with females, except the gains in longevity were not as pronounced in the males that were housed separate from females. Neither females nor males housed in vials had any hint of a mortality plateau, as was observed with T and CT flies housed in cages.

Tables 5 and 6 summarize the vial fly data from Fig 3. Table 5 shows the mean life span of *Drosophila* in days for flies housed in vials with single sex or combined sexes and with or without SC100. All SC100 treated flies have significantly different life spans from non-treated Controls (*P*<0.05%). Both males and females have significant longer life spans if they are housed in same sex vials than when housed with the opposite sex of 2 males with 2 females (*P*<0.05%).

**Table 5:** Mean *Drosophila* life span in days for flies housed in vials with the same or opposite sex: CA site.

**Fly Sample**	**Male**	**Female**
Control Combined	21.4	17.4
SC100 Combined	27.6	25.6
Control Single Sex	25.9	25.2
SC100 Single Sex	35.4	36.6

All SC100 treated flies had significantly different life spans from untreated Controls (*P*<0.05). Both males and females had significantly longer life spans if they were housed in same sex vials than when housed with the opposite sex (2 males with 2 females) (*P*<0.05). Each life span mean is based on the mean survival of 100 flies.

**Table 6:** Percentage Increase in Mean *Drosophila* Life span for Table 5 data samples compared.

**Samples Compared**	**Male**	**Female**	**Average**
SC100/Control Combined	29%	47%	38%*
SC100/Control Single	37%	45%	41%*
Control Single/Control Combined	21%	45%	33%*
SC100 Single/SC100 Combined	28%	43%	35%*

*All differences are significant (*P*<0.05) and are based on the mean survival of 100 flies.

Table 6 analyses the data in Table 5 and shows the increases in fly life span for various comparisons. As indicated from viewing the raw data in Fig 3, Table 6 shows that SC100 significantly enhanced the mean life span of flies housed in single sex vials (41% average increase on second row) and with both sexes combined (38% average increase on first row (*P*<0.05%). Females appeared to benefit more from SC100 than males, especially when both sexes were combined. The last two rows of Table 6 demonstrate that housing flies in single sex vials led to higher life spans than vials that combined the sexes (*P*<0.05%). Moreover, females had more improvement in life span in single sex vials than did males.

We have also done a Gompertz analysis of the effects of SC100 treatment on life span of flies housed in vials. Averaged over both sexes and both housing treatments (combined and separate), SC100 significantly increased the *A* parameter of the Gompertz model by 15% (*P* = 0.03). At the same time SC100 lowered the rate of aging parameters, α, by 36% (*P*<10^–8^). Overall, the more substantial decrease in α results in the observed increase in longevity.

### SC100 Did Not Extend the Long Life Span of *Drosophila* Housed in Vials under Low-Stress Conditions: AL Site

**Fig 4:**
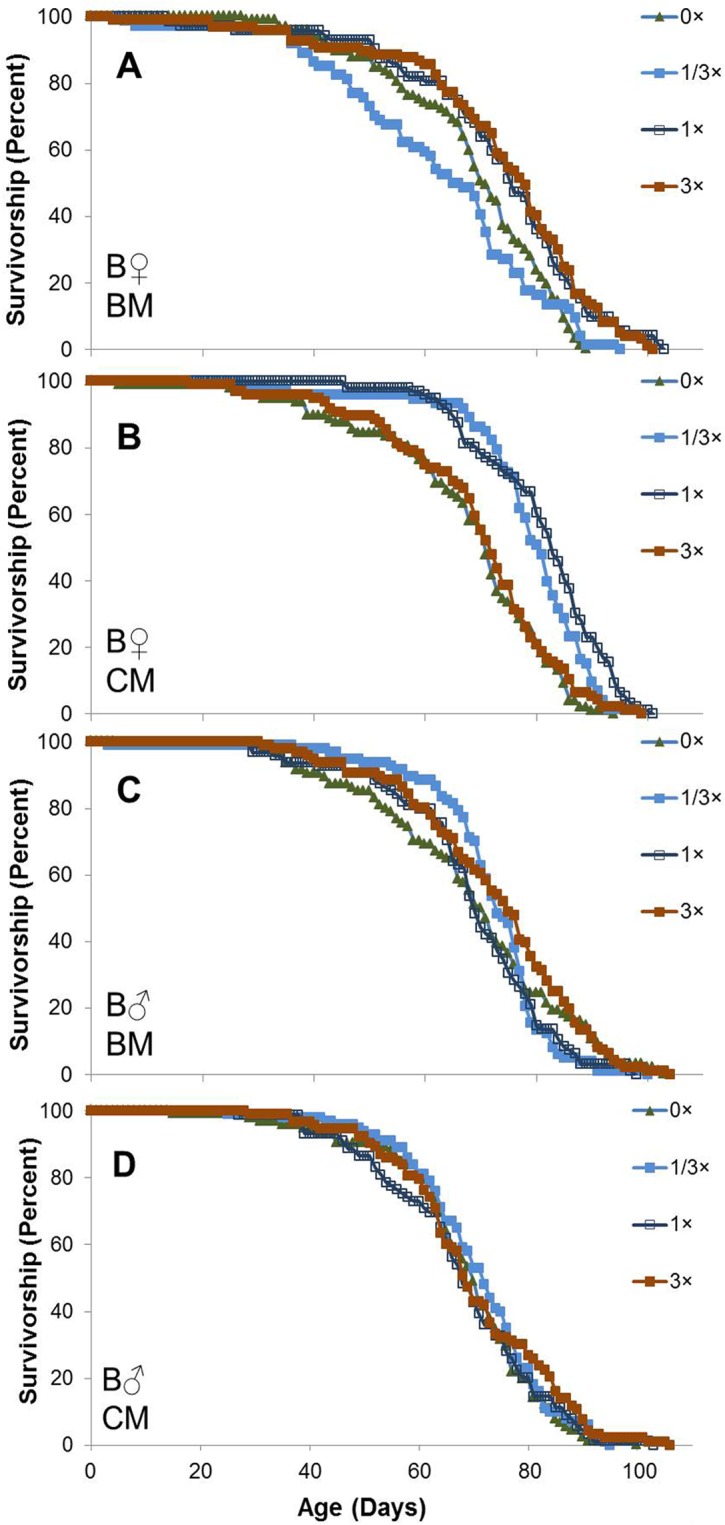
Life span of female and male B4 *Drosophila* in single sex vials with or without SC treatment: AL site. Flies were separated by sex 1 d after eclosion, housed in groups of 25/vial at 25°C on a 12 h light: 12 h dark cycle, and supplemented with SC100 at doses of 0× (solid green triangles), 1/3× (solid blue squares), 1× (open black squares), and 3× (solid red squares) beginning 2 d after collection. Results are presented separately for females (**A**,**B**) and males (**C**,**D**) on banana medium (BM; **A**,**C**) and cornmeal (CM; **B**,**D**). For each medium and dosage, n = 3–4 vials (72–98 surviving flies) for females and 4 vials (89–100 survivors) for males. Results presented in Figs 4–6 were obtained concurrently.

**Fig 5:**
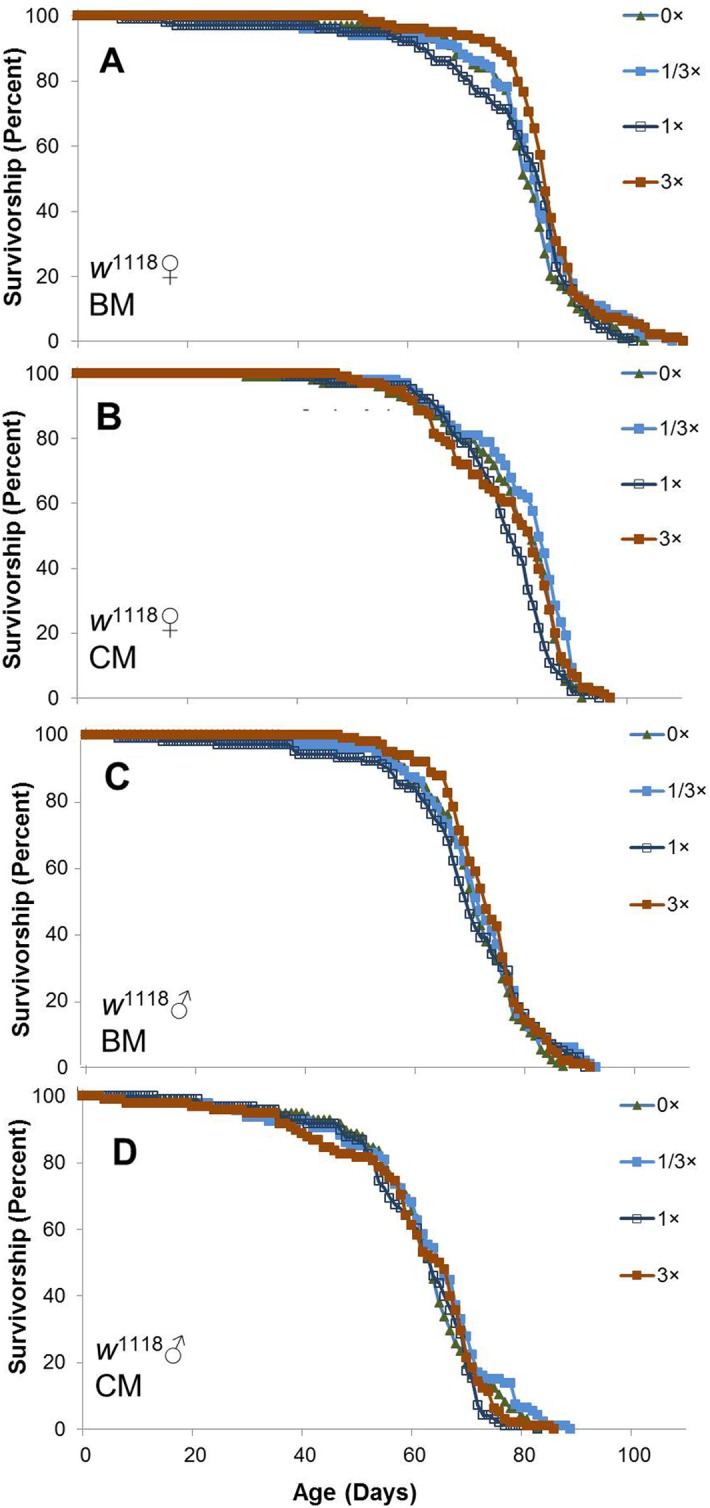
Life span of female and male *w* ^1118^ *Drosophila* in single sex vials with or without SC treatment: AL site. Flies were separated by sex 1 d after eclosion, housed in groups of 25/vial at 25°C on a 12 h light: 12 h dark cycle, and supplemented with SC100 at doses of 0× (solid green triangles), 1/3× (solid blue squares), 1× (open black squares), and 3× (solid red squares) beginning 2 d after collection. Results are presented separately for females (**A**,**B**) and males (**C**,**D**) on banana medium (BM; **A**,**C**) and cornmeal (CM; **B**,**D**). For each medium and dosage, n = 4 vials (96–102 surviving flies for females and 95–100 for males). Results presented in Figs 4–6 were obtained concurrently.

**Fig 6:**
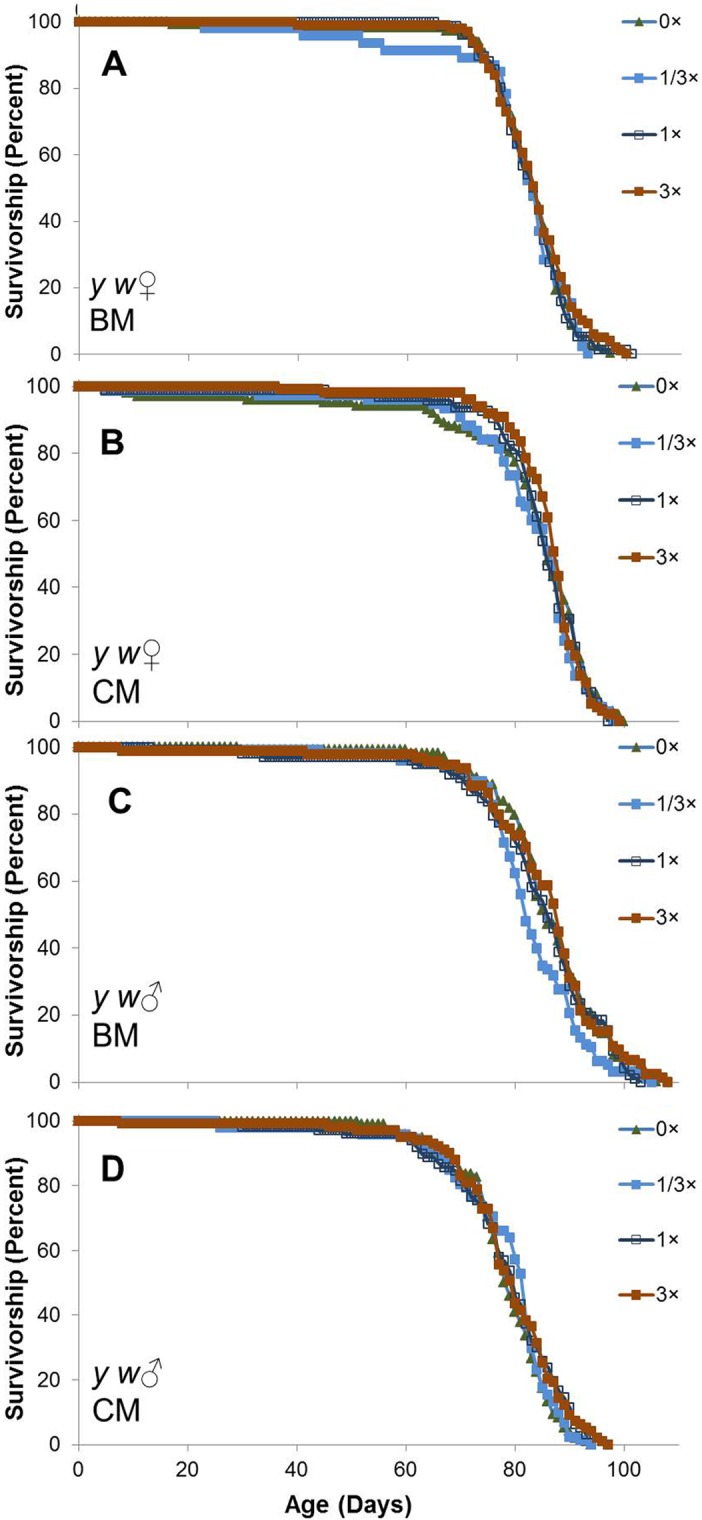
Life span of female and male *y w Drosophila* in single sex vials with or without SC treatment: AL site. Flies were separated by sex 1 d after eclosion, housed in groups of 25/vial at 25°C on a 12 h light: 12 h dark cycle, and supplemented with SC100 at doses of 0× (solid green triangles), 1/3× (solid blue squares), 1× (open black squares), and 3× (solid red squares) beginning 2 d after collection. Results are presented separately for females (**A**,**B**) and males (**C**,**D**) on banana medium (BM; **A**,**C**) and cornmeal (CM; **B**,**D**). For each medium and dosage, n = 3–4 vials (75–102 surviving flies) for females and n = 4 vials (91–99 survivors) for males, except n = 2 vials (46 survivors) for females on 1/3× BM (**A**). Results presented in Figs 4–6 were obtained concurrently.

To establish the consistency of effects of SC100 under different strain, food, and housing conditions, the supplement was tested by a separate group of investigators at an independent test site in Alabama (AL site) using low stress (single sex and 12 h on/12 h off lighting) vial conditions. SC100 was administered simultaneously to both female and male flies of three different genotypes provided with two different media, at three doses spanning a 9-fold range (1/3×, 1× and 3×). Flies housed in vials at the AL site exhibited highly rectangular survival curves, with no hint of a mortality plateau at advanced ages (Figs 4–6). Despite the 6 fold higher population density of flies in AL, the mean life spans of untreated (0×) flies were substantially longer for all groups in AL than CA, by factors of 2.7 for B females (68.7 *vs*. 25.2 d) and 2.6 for B males (68.1 *vs*. 25.9 d) on the same BM medium used in vials in CA. On the other hand, the maximum life span (90–100% mortality) was substantially shorter for supplemented flies in vials in AL than in cages in CA.

A factorial analysis of variance for mean life spans of all flies revealed significant differences based on sex, strain and food medium, but little or no difference among SC100 supplementation groups (Table 7). Significant interactions were observed for sex × strain, sex × medium, strain × medium and medium × SC100 dosage, but supplementation did not interact significantly with either sex or strain and none of the higher-order interactions were significant. Pooling across dosages of SC100 (0–3×), female *w* ^1118^ flies lived 16% longer than males on BM and 28% longer on CM. The sex differences were much smaller for the other strains: 0% on BM and 7% on CM for B4 females, and -3% on BM and 7% on CM for *y w* flies. Mean life spans of *y w* males were 20–27% longer than those of *w* ^1118^ males and 13–21% longer than B males on both media. *y w* females lived 14–18% longer than B females, but only 1–6% longer than *w* ^1118^ females. BM also yielded differences in life span ranging from 5% shorter to 15% longer than CM, depending on sex and strain. Pooling across all levels of sex, strain and medium, the overall effects of 1/3×, 1× and 3× SC100 on mean life span (relative to 0× controls) trended slightly upward at +0.8%, +1.2% and +2.4%, respectively, but these differences were not significant (*P* = 0.2, Table 7). Distributions of survivorship suggest that similar conclusions would be reached in comparisons of mean or maximum life spans.

**Table 7:** Effects of Sex, Strain, Food Medium and SC100 Supplementation: AL site.

**Source of Variation**	**SS** *	**df** *	**Mean Squares**	**F Ratio**	***P* Value**
Sex	1,637.405	1	1,637.405	97.793	**<0.0005**
Strain	4,257.391	2	2,128.695	127.135	**<0.0005**
Medium	195.453	1	195.453	11.673	**0.001**
Dosage of SC100	79.951	3	26.650	1.592	0.194
Sex × Strain	1,590.953	2	795.477	47.509	**<0.0005**
Sex × Medium	510.750	1	510.750	30.504	**<0.0005**
Sex × Dosage	99.736	3	33.245	1.986	0.119
Strain × Medium	469.642	2	234.821	14.025	**<0.0005**
Strain × Dosage	190.139	6	31.690	1.893	0.086
Medium × Dosage	204.352	3	68.117	4.068	**0.008**
Sex × Strain × Medium	15.656	2	7.828	0.468	0.628
Sex × Strain × Dosage	169.038	6	28.173	1.683	0.130
Sex × Medium × Dosage	69.309	3	23.103	1.380	0.252
Strain × Medium × Dosage	125.357	6	20.893	1.248	0.286
Sex × Strain × Medium × Dosage	156.206	6	26.034	1.555	0.165
Error	2,293.864	137	16.744		

*SS = sum of squares, df = degrees of freedom.

To establish whether the interaction of medium × SC100 dosage might reveal any effect of the supplement on longevity on one medium but not the other, a separate 3-way ANOVA was performed for each medium. For BM, differences were observed between the sexes and among strains and SC100 dosages (Table 8). Sex × strain interaction was also significant. Therefore, results for females and males were compared separately. In females on BM, there were differences among strains and dosages, but no interaction (Table 9); however, a 1-way ANOVA with strain as a covariate showed no difference among dosages (Table 10). In males on BM, there were differences among strains, but SC100 dosage had neither a main effect on life span nor an interaction with strain (Table 11). For CM, sex, strain and sex × strain interaction were significant; SC100 dosage had no effect on life span, but strain × SC100 interaction was significant (Table 12). Testing within each strain on CM, SC100 dosage had no main effect on life span or interaction with sex in *w* ^1118^ or *y w* flies, but SC100 dosage × sex interaction was significant for B flies (Table 13). Testing within each sex revealed differences among dosages for B4 females (*P* = 0.025), but not males (*P* = 0.8). Pairwise comparisons among dosages showed that supplementation with 1× SC100 extended life span by 20% in relation to unsupplemented controls (*P* = 0.040; Table 14).

**Table 8:** Effects of Sex, Strain and SC100 Dosage—BM Food: AL site.

**Source of Variation**	**Sum of Squares**	**df** *	**Mean Squares**	**F Ratio**	***P* Value**
Sex	155.489	1	155.489	8.967	**0.004**
Strain	2,560.046	2	1,280.023	73.817	**<0.0005**
Dosage of SC100	185.111	3	61.704	3.558	**0.019**
Sex × Strain	829.741	2	414.870	23.925	**<0.0005**
Sex × Dosage	69.737	3	23.246	1.341	0.269
Strain × Dosage	103.094	6	17.182	0.991	0.439
Sex × Strain × Dosage	123.843	6	20.640	1.190	0.322
Error	1,161.815	67	17.341		

*df = degrees of freedom.

**Table 9:** Effects of Strain and SC100 Dosage—Females on BM Food: AL site.

**Source of Variation**	**Sum of Squares**	**df** *	**Mean Squares**	**F Ratio**	***P* Value**
Strain	1,380.741	2	690.371	42.622	**<0.0005**
Dosage of SC100	179.438	3	59.813	3.693	**0.022**
Strain × Dosage	161.840	6	26.973	1.665	0.163
Error	502.122	31	16.197		

*df = degrees of freedom.

**Table 10:** Effect of SC100 Dosage with Strain as Covariate—Females on BM Food: AL site.

**Source of Variation**	**Sum of Squares**	**df** *	**Mean Squares**	**F Ratio**	***P* Value**
Dosage of SC100	143.702	3	47.901	1.781	0.167
Strain	1,023.200	1	1,023.200	38.039	**<0.0005**
Error	1,022.158	38	26.899		

*df = degrees of freedom.

**Table 11:** Effects of Strain and SC100 Dosage—Males on BM Food: AL site.

**Source of Variation**	**Sum of Squares**	**df** *	**Mean Squares**	**F Ratio**	***P* Value**
Strain	2,148.484	2	1,074.242	58.622	**<0.0005**
Dosage of SC100	62.106	3	20.702	1.130	0.350
Strain × Dosage	60.388	6	10.065	0.549	0.767
Error	659.693	36	18.325		

*df = degrees of freedom.

**Table 12:** Effects of Sex, Strain and SC100 Dosage—CM Food: AL site.

**Source of Variation**	**Sum of Squares**	**df** *	**Mean Squares**	**F ratio**	***P* value**
Sex	2,042.319	1	2,042.319	126.286	**<0.0005**
Strain	2,153.364	2	1,076.682	66.576	**<0.0005**
Dosage of SC100	98.841	3	32.947	2.037	0.117
Sex × Strain	772.630	2	386.315	23.888	**<0.0005**
Sex × Dosage	98.997	3	32.999	2.040	0.116
Strain × Dosage	218.356	6	36.393	2.250	**0.048**
Sex × Strain × Dosage	205.130	6	34.188	2.114	0.062
Error	1,132.049	70	16.172		

*df = degrees of freedom.

**Table 13:** Effects of Sex and SC100 Dosage—B4 Flies on CM Food: AL site.

**Source of Variation**	**Sum of Squares**	**df** *	**Mean Squares**	**F ratio**	***P* value**
Sex	186.277	1	186.277	5.972	**0.023**
Dosage of SC100	262.206	3	87.402	2.802	0.063
Sex × Dosage	285.597	3	95.199	3.052	**0.049**
Error	717.434	23	31.193		

*df = degrees of freedom.

**Table 14:** Tukey’s Honestly Significant Difference Test—B4 Females on CM Food: AL site.

**Dose (i)**	**Dose (j)**	**Difference**	***P* Value**
0× SC100	1/3× SC100	-11.070	0.139
0× SC100	1× SC100	-13.534	**0.040**
0× SC100	3× SC100	-1.910	0.969
1/3× SC100	1× SC100	-2.464	0.950
1/3× SC100	3× SC100	9.160	0.256
1× SC100	3× SC100	11.624	0.083

### Fecundity: CA Site

Dietary restriction and many genetic changes that extend life span typically have the unwanted side effect of depressing fecundity. To check whether this was the case with SC100 treatment, we carried out fecundity experiments with control and SC100-treated flies (Table 15). Two ages were tested: 2 weeks (young) and 4 weeks (old). The mean fecundity from a linear model analysis is shown in Table 15 for young and older Control and SC100-treated flies. There was no significant difference in fecundity for young females between Control and SC100-treated flies (*P* = 0.74). However, for older flies, SC100-treated flies had significantly higher fecundity than did Control flies (*P*<0.001). Thus, rather than inhibiting reproduction, these results indicate SC100 may actually preserve higher fecundity over control levels in later life.

**Table 15:** Mean fecundity estimated from a linear model of fecundity with two fixed effects: adult age and treatment.

**Age/Treatment**	**Fecundity**
2–4 week Control	23.8
2–4 week SC100-Treated	24.6*
4–6 week Control	13.3
4–6 week SC100-Treated	19.1*

Young Control and SC100-treated flies were tested for fecundity starting at 2 weeks old, whereas older Control and SC100-treated flies were fecundity tested at 4 weeks of adult age. Fecundity value is shown as average eggs per fly over a 2 week test period under optimal conditions.

*The 2–4 week SC100 Treated flies were not significantly different from Control flies (*P* = 0.74), whereas the 4–6 week treated flies were significantly more fertile than 4–6 week Control flies (*P*<0.001), but not significantly different from the younger 2–4 week Control flies in fertility.

### Heat and Starvation Stress Resistance on B4 and stress resistant O strains: CA Site

Genetic or environmental treatments that enhance life span also typically enhance resistance to environmental stress [1–3,5,6]. We first tested whether SC100 treatment also enhances stress resistance to partial starvation (Table 16) using B4 flies. Two hundred flies (100 flies of each sex) were placed in cages that had 10% of the normal food concentration at 25°C, but were otherwise unrestricted in food availability. This condition puts a starvation stress on the flies that leads to higher daily mortality rates. In two separate experiments (Table 16) the 50% median B4 fly survival fell to around 9–10 days with partial starvation. SC100 treatment enhanced stress resistance to starvation and led to flies with a 50% median survival of 15 to 17 days or about a 62% increase in median survival (*P*<0.001).

**Table 16:** Effects of SC100 on resistance to starvation stress.

**Treatment**	**Male 50% Survival**	**Female 50% Survival**	**Male Change**	**Female Change**
Exp. 1: Control	9.0 days	9.1 days		
Exp. 1: SC100-treated	15.0 days	15.3 days	67%*	68%*
Exp. 2: Control	10.4 days	10.7 days		
Exp. 2: SC100-treated	16.7 days	16.7 days	61%*	56%*

100 males and 100 females in each cage were subjected to 10% of normal food in Control or SC100-treated cages. The number of dead flies was monitored daily and the 50% median survival in days is given for both males and females. The effects of SC100 are shown as the increase in survival (Male or Female Change).

*In Experiments 1 and 2, survival times of treated flies were significantly different from those of Control flies (*P*<0.001).

**Fig 7:**
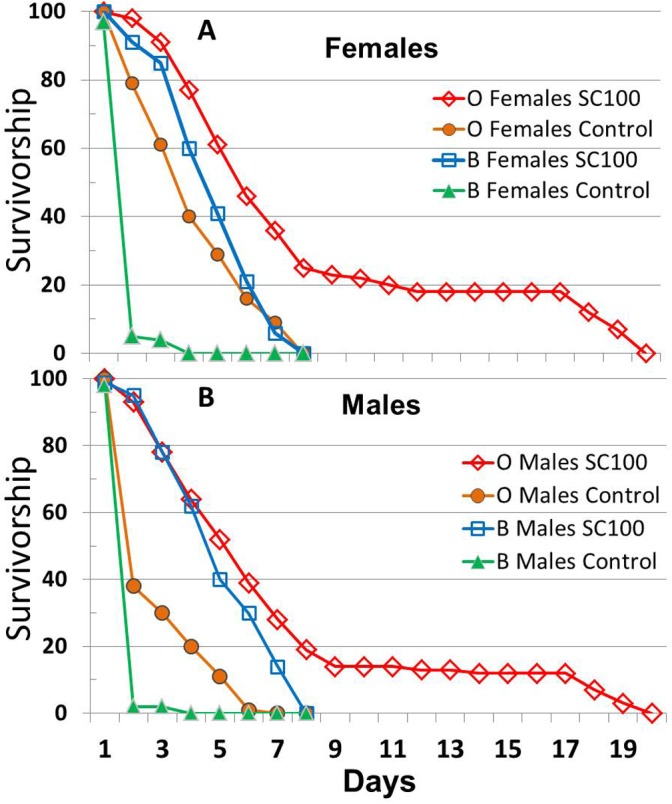
Survival curves for female and male B4 and O *Drosophila* in cages during heat and partial starvation treatment: CA site. 7A (Females) and 7B (Males) show the survivorship curves for B4 and O1 flies under heat (29°C) and partial starvation (10% dilution of normal food supply) stress. B4 Controls (solid green triangles) and stress-resistant O1 Control (solid red circles) flies were each housed in separate cages with both sexes combined (250 females and 250 males). Survivorship curves for the SC100 treated flies are shown for B4 treated (blue open squares) and for stress resistant O1 (open red diamonds) flies.

We have also tested whether SC100 treatment enhances stress resistance to a combination of heat and partial starvation using both B4 flies and the stress resistant Methuselah O1 fly strain (Fig 7). Female and male 20% B4 fly survival fell to less than 1 day with 29°C heat and partial starvation (green filled triangles). SC100 treatment dramatically enhanced stress resistance to the heat and starvation stressed B4 flies and led to a 20% survival of 4.7 days for females and 4 days for males (open blue squares).

With the much more stress resistant Methuselah O1 strain of *Drosophila*, the 20% survival time for the untreated O1 flies was 6.0 days for females and 6.6 days for males (Fig 7, orange solid circles). SC100 treatment of the O1 flies led to a 20% survival of 11 days for females and 8 days for males (Fig 7, open red diamonds). The SC100 treated O1 flies also had a dramatic increase in survival of the last 15–20% of the survivors (Fig 7, see long tail of open red diamonds in both males and females).

## Discussion

Results of this study show that an herbal supplement, SC100, can have strikingly divergent effects on the life span of *Drosophila* under different test conditions. In relatively high stress environments associated with short mean life spans, SC100 increased mean and maximum survival times in both cages and vials and increased resistance to partial starvation stress without decreasing fecundity. In cages, it was also associated with a highly distinctive distribution of survivorship, with only a slight effect on early mortality and mean longevity but a vast impact on late mortality and maximum longevity. In vials, SC100 was beneficial to the entire population under high stress conditions, postponing both early and late mortality. In a lower stress environment, it had no overall effect on survivorship, but it was associated with a longer mean life span in female flies of the B4 strain on one food medium.

In comparing the vial experiments in CA to the vial experiments in AL, the mean life span for the untreated B4 flies in single sex vials in CA (25.9 d) was much shorter than the untreated single sex vials in AL (68.7 d). These very large increases in the life spans of untreated flies at the AL site are likely due to environmental differences between the CA and AL sites, resulting from a low stress environment at the AL site. However, housing flies in cages (CA site) strongly promoted longevity in a subfraction of the SC100-treated flies, leading to flies that had a maximum life span that exceeded the maximum life span found for any of the treated or untreated flies at both the CA and AL sites.

### Altering the Survival Curve

While the ultimate causes of aging are still in dispute, aging can be simply defined as the biological changes that lead to progressive age-related increases in mortality rates. As an animal cohort ages, survival has often been expressed by the well-known Gompertz curve, wherein the number of individuals alive in the population declines due to an exponential increase in mortality rates with age. For example, the Gompertz survival curve fits human populations quite well for ages of 25 to 85 years [45] and is driven by the exponential increase in the annual mortality rate or risk of dying with age. In humans, the annual mortality rate doubles every 8 years. As human survivors over age 85 have increased in recent years, the Gompertz curve has become a little less accurate in predicting mortality rates at extreme ages. Specifically, older humans are living slightly longer than predicted by the Gompertz curve. Moreover, the very rare humans that survive to the supercentenarian status of 110 years reach an apparent plateau in annual mortality rate of some 50 to 65% per year [46], suggesting that this highly selected group of supercentenarians may deviate from the standard Gompertz curve dynamics. One explanation of this is that the highly selected supercentenarians have a more favorable genetic background for longevity and therefore have different Gompertz parameters (especially alpha).

While the Gompertz curve with its exponential increases in mortality rates with age was originally proposed in 1825 for human populations, it has also been highly successfully in modeling mortality rates in aging populations of many animals [47]. Similar to the case with human survival curves, data from several groups began appearing in the 1990s indicating that fruit flies, medflies, nematodes, wasps, and yeast may hit a mortality rate plateau at later ages, with only a very small subfraction of 1% of the population cohort still alive [48]. Thus, like humans, it appears that the parameters of the Gompertz curve may be altered in a small subfraction of the oldest members of an animal cohort.

Of the successful interventions to change animal survival curves (i.e. life span), transgenic animal mutants and dietary restriction have typically generated the best results [49,50]. However, many genetic mutations and even dietary restriction have now been shown to extend life span to various extents depending on the variable experimental conditions [51–57]. This variability in life extension is consistent with our results in the present paper: the life span extension of SC100 depends on the exact genotype, diet, and laboratory conditions. Stress resistance or a low stress environment appears to be an underlying factor, as those conditions that lower stress favor longer life spans [3].

Successful interventions into aging have been detected as increases in mean and/or maximum life span. Interventions that largely affect mean rather than maximum life span are often observed to “square the survival curve” and to affect healthspan more than life span or aging. Indeed, some researchers believe that only treatments that increase maximum life span are actually impacting the rate of aging. However, we are not aware of any interventions, whether genetic or environmental, that double fly maximum life span and effect such significant changes in the alpha parameter of the fly Gompertz curve. We clearly show in this article that treatment of caged flies with SC100 alters the alpha parameter of Gompertz survival curve in a fundamental way.

### Decline in Late-Life Mortality Rate

While some previous published articles have reported a decline in late-life mortality rate in a small fraction of 1% of cohort survivors [48,58], our fly data indicate that SC100 induces a greatly reduced mortality rate in late life at around 33% fly survival. Unlike worms and yeast, genetic and dietary interventions have been limited in their capacity to extend mean and maximum life span in *Drosophila* by more than 50%. The elongation of maximum life span of caged flies by SC100 appears to outperform the previously published *Drosophila* life span enhancing treatments using transgenics and dietary restriction methods. We have also shown that SC100 treatment generates robust resistance to starvation and heat stress (Table 16, and Fig 7).

In the case of dietary restriction and many treatments that extend life span, there are often trade-offs such as reductions in overall egg production and fertility [59,60]. It appears that reducing egg production promotes longer life span. In the present longevity experiments, fecundity was the same initially in SC100 treated and control flies, but became significantly higher in treated flies compared to controls as the control flies aged. Thus, SC100 appears to have striking effects on reducing both aging and mortality rates without the typical negative effect on fecundity. Measurements of walking speed, duration of flight, spontaneous locomotor activity, oxygen consumption or carbon dioxide production would be needed to rule out trade-offs entirely, but none were apparent to visual inspection of the caged flies.

The main cause of the large increase in maximum life span of caged flies produced by SC100 appears to be the generation of a large decline in the late-life mortality rate after some two-thirds of the population has died. In previous experiments where major declines in the late life mortality rate have been reported, they occurred in only a vanishingly small fraction of 1% of survivors from very large populations [48,58,61]. In our current *Drosophila* experiments using cages, it is striking that SC100 treated flies reach a much reduced mortality rate when roughly one third of the flies are still alive and the daily mortality remains fairly low. Even when we started treating flies at midlife, when about 40% of the cohort had died, the remaining flies reached a much reduced mortality rate when roughly a third of the flies were still alive. Note that under these conditions with the cohort population of 1500 flies, we only see a ragged mortality rate decline in the control flies among females beginning when fewer than 10% of the flies were still alive and only lasting for a few days (Fig 2C). Thus, the SC100 treatment dramatically altered the aging of *Drosophila* and apparently did so by preventing significant increases in the mortality rate for an extended period of time in a substantial fraction of the population. If a treatment could generate a significant decline in the mortality in mammals while a large part of the population was still alive, that could be a novel way of extending longevity in the surviving fraction of the population.

### Effect of SC100 on Longevity Depends on Environmental Conditions

Vast phylogenetic, anatomical and physiological gaps separate *D*. *melanogaster* and *Homo sapiens*. Therefore, the consistency of effects of SC100 on fly longevity was assessed under distinctly different environmental conditions as a minimal precondition for any speculative inferences regarding human aging. Experiments were performed independently in cages and in vials at different population densities, with males and females segregated or housed together. A different rationale underpinned each set of conditions. First, while most *Drosophila* longevity experiments in the literature are still carried out using only one sex in either vials or bottles, large Plexiglas cages with 250 flies of each sex provide a more natural environment that does not prevent reproduction or exercise in the form of sustained flight. On the other hand, flies may differ from humans in that both mating and exercise (increased metabolic activity) typically shorten the life span. Nevertheless, a surprising finding in our results was that the cage experiments containing both sexes where flies were frequently flying nevertheless had the longest maximum life spans.

As has been found in many other laboratories, mean longevity was markedly lower in this study when males and females were mixed in large cages than when they were housed separately in small vials at the CA or AL sites. SC100 was associated with a strong enhancement of the short mean life spans in vials and cages at the CA site and almost no effects on long life spans in vials at the AL site. Surprisingly, even though the population density was lowest in vials at the CA site, these flies exhibited even shorter mean and maximum life spans than the control flies in cages. Assuming that the length of life in control flies is indicative of the intensity of stress, SC100 supplementation was beneficial to the entire population under conditions of maximum stress (vials at the CA site), a large subpopulation at intermediate stress (cages at the CA site) but not to any of the flies under low stress (vials at the AL site). Of course, this interpretation does not identify the source of stress in the vials at the CA site. However, we do note that a 12 h light: 12 h dark cycle was used at the AL site, whereas the lights were on 24 h every day for all experiments done at the CA site. Disruption of the circadian clock has been shown to significantly shorten life span in Drosophila [62], so this is one stress factor that we can identify that could account for some of the difference between mean life spans in vials at the AL versus CA sites.

### SC100 as a Multipath Approach to Enhancing Stress Resistance and Slowing Aging

How is SC100 acting and do the fly housing conditions matter? SC100 could be extending life span at the CA site by inducing more repair, autophagy, or regenerative capacity. These pathways were targeted, but we do not have direct evidence that there were significant changes in these genetic pathways to explain the life extension at this site. We also had differences in the survival curves depending on whether we used large cages with 500 flies or small vials with 4 flies in each vial (compare Fig 1 to Fig 3) or very low stress vials under near ideal conditions. This shows that major differences in housing conditions for the flies can lead to major differences in survival curves. Of course, it has been known for some time that housing flies in a same sex environment extends life span, but we have now shown that differences in survival are also observed if one uses cages with large populations versus small vials. We have also obtained very long mean life spans of over 70 days with untreated wild-type same-sex B4 *Drosophila* in vials housing 25 flies per vial under ideal low stress conditions. In those cases where the controls were in low stress housing conditions with normal circadian rhythm, SC100 had little or no life-extending effect. These experiments suggest that fly housing can greatly alter life span survival curves and the effects of supplements can vary depending on housing. Our hypothesis is that a favorable low stress environment strongly promotes longevity in control flies.

Is SC100 acting only on a genetic subset of the flies (the cohort heterogeneity model)? While we cannot completely rule out this possibility, it seems highly improbable that one third of an interbreeding *Drosophila* population could have a strikingly different genotype or gene expression pattern, as this B4 population of flies has been interbreeding for some 30 years. The evolutionary theory of aging could provide an explanation for our results if we assume that SC100 induces an alternative pattern of gene expression that counteracts dysfunctional gene expression patterns that develop at later fly ages, leading to an alteration in the Gompertz age-related alpha parameter and an elongated survival curve.

It is safe to say that genetics, living environment, and diet are all factors determining longevity. Indeed, longevity appears to be quite plastic and responsive to many factors. We have focused mainly on changing the diet using multiple dietary supplements. The many active components in SC100 may provide a multipath approach to slowing aging and to altering the basic age-related alpha parameter of the Gompertz curve. Our data in *Drosophila* housed in cages indicate that such an approach may provide a practical way to dramatically slow the aging process in this important model animal. We are not aware of any treatment with a mix of substances or any of the many single-gene transgenics that has had more than a 50% increase in *Drosophila* maximum life span, so the effects of SC100 on *Drosophila* maximum life span in our cage experiments are impressive. *Drosophila* treated with SC100 underwent a significant alteration in their alpha parameter and thus slowed their rate of aging. However, these results should be taken with the caveat that housing environment and stress can also be a big factor in life span. The ability of housing environment alone to generate long-lived flies (mean life span of >70 days for control flies, as was the case in the low-stress, same-sex vial experiments) suggests that longevity gains can be promoted by alternative methods in non-synergistic ways.

Concerning ramifications for humans, two opposite interpretations can be entertained. (1) Reducing stress and promoting stress resistance both appear to be valuable interventions to extend life span. With modern life apparently becoming more stressful, environmental interventions appear to be an effective approach to promoting health span and life span. (2) For readers in developed nations with historically unprecedented rectangularization of survivorship, where many bacterial and viral illnesses have been eradicated, where safe water, food, waste disposal, and medications are readily available, and where life can be spent in thermally controlled indoor environments, escaping strenuous physical activity, the results from the AL site suggest that SC100 might not lead to further extension of life, unless the individual had a stressful job, did not get enough sleep, or made unhealthy lifestyle choices. For those people with stressful lives, the results from cages suggest that a long-lived subpopulation could benefit substantially from SC100 supplementation. Under exceptionally stressful conditions, the results from vials at the CA site suggest that supplementation would assist the whole population.

A broader point is that realistic inferences about human supplementation should await replication of the results in mice and/or data from human clinical trials. A version of SC100 is currently being testing in a double-blind, placebo controlled clinical trial.

## Materials and Methods

### Testing Sites and Data Availability

Longevity experiments were performed at two sites: Genescient Inc., Fountain Valley, CA (designated ‘CA’) and the University of South Alabama, Mobile, AL (designated ‘AL’). Fertility and stress resistance were measured at the CA site. All relevant data is within the paper.

### Fly Strains and Media

All studies at the CA site employed stocks of the wild-type B4 line or the late-fertility-selected O1 line of *D*. *melanogaster* (originally from the Michael Rose laboratory at the University of California, Irvine), which were maintained in vials containing a banana medium at about 25°C. The banana medium (BM) is a *low protein* diet containing a boiled mixture of (i) 100 g agar in 6.6 L distilled water, (ii) 900 g peeled bananas, 165 mL Eden barley malt syrup, 110 mL light and 110 mL dark Karo corn syrup blended into 400 mL distilled water, and (iii) 240 g Fleischmann’s instant dry yeast and 160 mL 95% ethanol blended into 460 mL water. After cooling to 48°C, 15.6 g methyl-4-hydroxybenzoic acid (10% w/v in 95% ethanol) was added as an antifungal agent. Life spans on this medium are nearly as long as on dietary restriction diets [63]. Studies at the AL site employed the B4 line, which was shipped from the CA site and allowed several weeks to acclimate, and parallel groups of flies from *y w* and *w* ^1118^ strains. Stocks were maintained on a sucrose/yeast/cornmeal diet described previously [55].

### Drug/Food Preparation

The SC100 herbal drug is a proprietary 4 herb blend comprising: 1) medicinal Astragalus membranaceus extract; 2) Pterocarpus marsupium standardized for 25% pterostilbene; 3) Pine Bark extract standardized for 85% Oligomeric-Proanthocyanidins, and 4) 98% pure L-Theanine. SC100 was obtained from Life Code, Inc. (contact info@LifeCodeRx.com for SC100 research samples or purchase the commercial version of SC100 online at www.lifecoderx.com).

For studies in CA, the SC100 dosage was calculated and prepared by pharmacist Marc Horwitz and independently verified. The standard dilution of SC100 (5% w/w) has a human dose equivalent equal to 1 mL of the sample. The food plates were prepared using a syringe to measure out the appropriate SC100 dosage and transferred to a small “salsa dish” on a Denver Instrument XL-410D fine scale with an accuracy of +/- 0.001 g. The SC100 was then diluted with BM at 40–60°C up to 10.00 g (+/- 0.01 g). The food plates containing SC100 were labeled “T” or “CT” and the control plates were labeled “C”. Food was prepared every 2–3 d and was never kept for more than 3 d to ensure freshness. The dilution was re-made weekly and stored at 4°C.

For studies in AL, the dosage was calculated based on a 10^8^ fold difference in mass between flies and humans (0.7 mg *vs*. 70 kg) and ingestion of 2 μL food per fly per day [64]. Accordingly, a concentration of 4.5 mg/L SC100 (1X) corresponded to 9 ng per fly per day, equivalent to a human dose to 900 mg or 2 capsules per day. SC100 was diluted 1:10 from stock solutions into both BM and cornmeal medium (CM) at doses of 0 (control), 1/3X, 1X and 3X during cooling (40–60°C). Fresh food was prepared every 2–3 d and stored at 4°C. Fresh stock solutions were prepared weekly and stored at 4°C.

### Fly Longevity Assays—Cages

All assays in cages were performed at the CA site. Flies were separated into 9 groups of 250 males and 250 females per cage. Thus, there were a total of 9 cages in the assay. Three cages of B4 population flies (T) were treated with 1 mL of SC100 solution (5% w/w) in 10 mL banana-agar media daily from day 1 after eclosion. Three cages of B4 population flies (CT) were treated without SC100 for 36 days before starting SC100 treatment with 1 mL of SC100 solution. Three control cages of B4 population flies (C) were given 10 mL banana-agar media daily without SC100.

### Fly Longevity Assays—Vials

As an alternative longevity assay, flies at the CA site were also housed in small vials with 4 flies/vial and enough vials to have 100 flies of each sex. In this assay, B4 flies were housed with 4 flies of the same sex in each vial or with 2 females plus 2 males per vial. The vials were changed every 2–3 d or 3 times per week, with flies combined into new vials to preserve the 4 flies per vial as flies died. Sexual selection (2 of each sex or all of one sex) was preserved until all the flies were dead.

At the AL site, male and female flies were housed separately in 4 groups of 25/vial. Vials were exchanged and mortality was recorded every 2 d initially and daily after age 40 d. In this case, the mean survival time of the original 25 flies was determined, so dead flies were not replaced to preserve the population density.

### Mortality Assay

At the CA site, mortality counts were conducted between 1–4pm every day. Dead flies were removed from the cages and scored along with gender data. Stocks of *Drosophila melanogaster* were cultured in polystyrene vials one quarter full of banana agar medium at an ambient temperature of 25°C. At 14 days of age from the egg (eclosion occurs 7 to 9 days from egg), the flies were sexed after being anesthetized and then transferred to Plexiglas cages and kept throughout adulthood at the same ambient temperature. The cages were custom made by Plastic Sales, Inc. in Costa Mesa, CA. The cages were made from 0.5 cm thick Plexiglas sheets to form a box 25 cm long, 20 cm wide, and 14 cm high. Cages were designed so that the flies cannot escape with daily feeding and the gathering of dead flies. Once in the cages, the flies were fed 15×100 mm Petri dishes full of banana agar with 5% dry yeast. In the SC100 cages, the yeast paste contained the assigned dosage (equivalent to a weight adjusted human dose of 820 mg) of SC100. The food dishes were changed daily until all the flies were dead.

When fly cohorts are maintained in population cages of the design that we used, eggs laid away from the food medium fail to develop successfully, while the food medium is removed sufficiently often that development cannot be completed when the eggs are laid there. Furthermore, the pupal stage required to complete development is of sufficient duration to make the development of offspring easy to detect; no such pupal development was observed in the course of these experiments.

We set up three cages for Control, lifelong SC100-treated, and midlife SC100-treated flies, including two sets of four control cages. Each cage contained 500 young, mature fruit flies of the species *D*. *melanogaster*. Every day we supplied the cages with new Petri dishes of banana-yeast agar media. We counted, sexed and recorded the number of dead flies in each cage daily. The dead flies were removed from the cages using an aspirator. Male flies were identified by the presence of sex combs on the forelegs and female flies were identified by their larger, striped abdomens and the absence of the sex combs. The few flies that died or escaped as a result of handling procedures were not recorded because they did not die as a result of ingestion of their assigned substance. The above procedure continued until all of the flies in all of the cages had died.

### Fecundity

For each of the lifelong and midlife SC100 treated samples, and the controls, we set up 60 vials, one quarter full of charcoal agar media, containing one treated female and one treated male (or untreated in the case of the controls.

We counted and recorded the number of eggs laid by the flies in each of 20 vials for each cage, 60 for each dose, after one day spent in the charcoal vials. We also conducted fecundity assays at 14 days and again at 28 days of cage life.

### Heat and Partial Starvation Stress Experiments

For the stress experiments we used survivorship curves for B4 and O1 flies under heat (29°C) and partial starvation conditions. Partial starvation used a 10% dilution of the normal banana-agar medium. For each of the 4 samples (Control and SC100-Treated B4 flies along with Control and SC100-Treated O1 flies), 250 flies of each sex were housed in cages as described above and survival was followed as before.

### Statistical Analysis

All statistical analyses of life spans at the AL site were performed as described previously [55]. In summary, the mean life span was calculated for each vial and the 4 means for each group were compared by analysis of variance, with sex, strain, medium and supplement dose as factors. Previous studies under similar conditions have shown that raw survivorship data do not violate parametric modelling assumptions (normal distribution and equality of variances) in most cases in this laboratory, and mean survivorship almost never violate the assumptions [55].

Data from the CA site were analyzed with R (R Development Core Team, 2011, version 2.13.1; see http://www.R-project.org/), as follows:

#### Mean Longevity

For each replicate cage and each sex the mean longevity was computed. Mean longevity (*l*_*ijk*_) was then modeled with the linear equation,

$$l_{ijk}=\alpha +\delta_{i}\beta_{i}+\delta_{j}\gamma_{j}+b_{k}$$

where δ_*i*_ = 0 if *i* = 1 and 1 otherwise, β_*i*_ measures the effect of treatment (control, CT, or T), γ_*j*_ measures the effect of sex (male or female) and *b*_*k*_ is the random contribution of block *k*, which is assumed to have a zero mean and variance of σ^2^. The parameters were estimated by the *lme* function in R—see Chapter 2 in reference [65] for a description of maximum likelihood methods used in the lme function.

#### Age-Specific Survival

In this formulation, we let the index *i* indicate one of the treatments (control, CT, T), *j* indicate sex (1 = female, 2 = male), *k* indicate a replicate cage, and *t* indicate age. Then the predicted probability of survival from the start of the experiment to age *t* was *y*_*ijkt*_. The basic nonlinear model is given by,

$$y_{ijkt}=f\left(\Psi_{ijk},t\right)+\varepsilon_{ijkt},$$ 1

where Ψ_*ijk*_ is the vector of parameters, *t* is the age, and *ε*_*ijkt*_ is the replicate cage variation. The function *f* is the Gompertz model,

$$f\left(\Psi_{ijk},t\right)=\text{exp}\left\{\frac{A_{ijk}}{\alpha_{ijk}}\left[1-\text{exp}\left(\alpha_{ijk}t\right)\right]\right\}.$$ 2

The parameter *A* is sometimes called the age-independent parameter of the Gompertz model and is a reflection of background mortality that does not change with age. On the other hand, α is called the age-dependent parameter and measures the rate at which mortality increases with age, e.g. senescence. We assume that the parameters of the Gompertz equation may be affected by the fixed effects, sex and drug treatment and the random cage environment. These assumptions translate into a system of equations,

$$\begin{array}{l} A_{ijk}=\beta_{1}+\gamma_{1i}\delta_{i}+\phi_{1}\delta_{j}+b_{1k}\\ \alpha_{ijk}=\beta_{2}+\gamma_{2i}\delta_{i}+\phi_{2}\delta_{j}+b_{2k} \end{array},$$ 3

where δ_*i*_ = 0 if *i* = 1, or 1 otherwise. To test for significant effects of SC100 treatment on *A* and α, we determined whether γ_1*i*_ or γ _2*i*_ was significantly different from zero, respectively. Likewise a test for the effects of sex on *A* and α corresponds to a test for whether ϕ_1_ or ϕ_2_ is significantly different from zero.

The variance of mortality is expected to change with the mean value of mortality. The general formulation for the variance of *ε*_*ijkt*_ is

$$Var(\varepsilon_{ijkt})≅\sigma^{2}g^{2}\left({\hat{u}}_{ijkt},t\right),$$ 4

where ${\hat{u}}_{ijkt}=E(y_{ijkt}|b_{i})$. In this analysis we used *g*(.) = | *y*_*ijkt*_|^δ^. The **b**_*i*_ were distributed as,

$$b_{i}∼N\left(0,\left[\begin{array}{cc} \sigma_{1} & 0\\ 0 & \sigma_{2} \end{array}\right]\right)$$ 5

The parameters in Equations (3–5) were estimated from a nonlinear mixed effects model as implemented by the *nlme* package of R—see Chapter 7 in reference [65].

### Female Fecundity

Fecundity was measured in females at two ages in all three SC100 treatments. We used a simple linear model to estimate the effects of age, treatment, and their interaction with the R linear model (lm) function. This linear model provides estimates of the magnitude of the effects of each drug and their statistical significance.

### Software

All statistical analyses in CA were carried out with R (version 2.7.0 and 2.7.1, The R Foundation for Statistical Computing). The Gompertz analysis of the supplements used the nonlinear, mixed effects R program (*nlme* R-package). The fecundity results were analyzed with the linear model function (*lm*) of R. The Gompertz utilized R-code used the *optim* R-function for finding maxima of the likelihood function. Analyses in AL were carried out using SYSTAT 12 software.
